# Treatment patterns and health outcomes in metastatic renal cell carcinoma patients treated with targeted systemic therapies in the UK

**DOI:** 10.1186/s12885-020-07154-z

**Published:** 2020-07-17

**Authors:** Robert Hawkins, Kate Fife, Michael Hurst, Meng Wang, Niroshini Naicker, Sarah Nolasco, Tim Eisen, Athena Matakidou, Jason Gordon

**Affiliations:** 1 University of Manchester and The Christie Hospital, Manchester, UK; 2 Cambridge University Hospitals NHS Foundation Trust, Cambridge, UK; 3 Health Economics and Outcomes Research Ltd, Cardiff, UK; 4 Bristol Myers Squibb Pharmaceuticals Ltd, Uxbridge, UK; 5 University of Cambridge, Cambridge, UK

**Keywords:** Renal Cancer, Molecular targeted therapy, Survival analysis

## Abstract

**Background:**

Patients with metastatic renal cell carcinoma (mRCC) treated with targeted systemic therapies have demonstrated favourable outcomes in randomised controlled trials, however real-world evidence is limited. Thus, this study aimed to determine the effectiveness of targeted systemic therapies for patients with mRCC in routine clinical practice in the UK.

**Methods:**

A retrospective, observational, longitudinal study based on chart review of newly diagnosed adult mRCC patients treated at two UK hospitals from 2008 to 2015 was conducted. Targeted systemic therapies recommended for use in mRCC patients were evaluated across first to third lines of therapy (1LOT-3LOT). Important exclusions were treatment with cytokine therapy and within non-standard of care clinical trials. Primary outcome measure was overall survival (OS); data were analysed descriptively and using Kaplan-Meyer analysis.

**Results:**

652 patients (65.3% male, 35.0% ≥70 years) were included. In 1LOT, 98.5% of patients received sunitinib or pazopanib. In 2LOT and 3LOT, 99.0 and 94.4% received axitinib or everolimus. Median OS was 12.9, 6.5 and 5.9 months at 1LOT, 2LOT and 3LOT respectively. Estimated OS at 1-year was 52.4% (95% CI: 48.6–56.4%) in 1LOT, 31.5% (25.2–39.5%) in 2LOT and 23.8% (10.1–55.9%) in 3LOT. Median OS from 1LOT in favourable, intermediate and poor MSKCC were 39.7, 15.8 and 6.1 months respectively.

**Conclusions:**

In this study, treatment was consistent with current National Institute for Health and Care Excellence (NICE) guidelines for mRCC patients. Although the study population favoured poorer prognosis patients, outcomes were more favourable than those for England at the same time. However, overall survival in this ‘real-world’ population remains poor and indicates significant unmet need for effective and safe treatment options to improve survival among mRCC patients.

## Background

Kidney cancer is the seventh most common cancer in the UK, accounting for 3% of all new cancer diagnoses [1]. Among all forms of kidney cancer, renal cell carcinoma (RCC) accounts for more than 80% of cases. Furthermore, 25–31% of UK patients have metastatic RCC (mRCC) at initial diagnosis. Five-year survival rates of RCC vary greatly depending on the stage of the disease, decreasing from 82 to 84% in patients with stage I disease to 5.2–6.6% in those with stage IV [1]. Recurrence rates are also significant, with up to 60% of patients who have undergone surgical intervention for localised disease experiencing a relapse within 5 years of nephrectomy [2]. Therefore, the number of patients requiring treatment for advanced and/or metastatic disease is substantial.

Cytokine therapy with interleukin-2 (IL-2) or interferon-alpha (IFN-α) had been the standard approach to treating mRCC patients for over 20 years. However, poor response rates, marginal survival benefit and significant toxicity [3] prompted the development of alternative treatment options for the majority of patients. As the understanding of the molecular biology underlying RCC has increased, specific molecular targets have been identified for potential therapies. These molecular-targeted therapies have improved efficacy and tolerability over cytokine therapy, and subsequently several targeted therapies have become available for first- and second-line use.

Targeted systemic therapies have been associated with significant improvements in progression-free survival (PFS) and, in some cases, overall survival (OS), better quality of life and a lower incidence of adverse events (AEs), compared to cytokine therapy [3–10]. However, outcomes from tightly controlled clinical trials are not always reflective of routine clinical practice, thus, there is a need to generate evidence on the real-world effectiveness of treatments for patients with mRCC.

This study aimed to determine the real-world effectiveness of treatments for patients with mRCC by describing the clinical characteristics and outcomes of patients treated with targeted systemic therapies in routine UK clinical practice based on contemporaneous data collected from two large hospitals.

## Methods

### Patients

A retrospective, observational, longitudinal cohort study based on a chart review was undertaken for newly diagnosed mRCC patients treated with systemic therapies at two UK hospitals (Addenbrooke’s Hospital, Cambridge and The Christie Hospital, Manchester). Patients were included if they were initially diagnosed with mRCC (index date) without concomitant malignant tumours between 01 January 2008 and 31 December 2015, were ≥ 18 years at the index date, had complete treatment information available and were followed up until death or the end of follow-up (31 December 2016). Patients were excluded if they satisfied one or more of the following criteria: a) not treated exclusively with NICE/cancer drug fund (CDF)-recommended systemic therapies that targeted receptors and signalling pathways involved in aspects of the cell cycle; (b) took part in a clinical trial in which the systemic therapies were not administered within standard of care (SoC); or (c) lost to follow-up (LTFU) prior to 30 June 2016. Patient consent was not required because this study was a retrospective chart review of anonymised data.

### Data collection

An electronic case report form (eCRF) was used to capture demographic and clinical data on eligible patients at baseline, including: age at index date; ethnicity; relevant comorbidities in the year prior to index date; Eastern Cooperative Oncology Group (ECOG); performance score (PS); Memorial Sloan Kettering Cancer Centre (MSKCC) risk score [11, 12] and histological subtype. Information on treatment patterns, including treatment prescribed in each line of therapy (LOT), the number (first/second/third) line of therapy (1LOT/2LOT/3LOT respectively), treatment initiation and discontinuation dates, reason for treatment discontinuation, treatment holiday, change in dose and AE incidence were gathered from medical records. The principal endpoint was OS, defined for each LOT as time from treatment initiation to death or most recent medical record. This was further stratified by treatment regimen, age, MSKCC score and histological subtype. All data were anonymised at a site level to ensure patient confidentiality before being sent for pooled analyses. The MSKCC risk score was re-calculated at this time to ensure across-site alignment.

The study complied with the International Society for Pharmacoepidemiology (ISPE) Guidelines for Good Pharmacoepidemiology Practices (GPP) [13] and applicable regulatory requirements. All required approvals from Ethics Committees, Independent Review Committees, Regulatory Authorities, and/or other local governance bodies were obtained.

### Data transformation

Only targeted systemic therapies recommended by the National Institute for Health and Care Excellence (NICE) for use in this patient population were evaluated. At the time of analysis (April, 2019), NICE supported the use of cabozantinib, tivozanib, pazopanib and sunitinib as first-line treatments (1LOT) in patients with advanced or metastatic RCC [14]. At second-line (2LOT), everolimus, axitinib, nivolumab, cabozantinib, and lenvatinib (combined with everolimus) were supported. Treatment pathways agreed by NICE indicate that approved 2LOT treatments are also used for third-line therapy (3LOT) [15].

To align with NICE-recommended pathways, patients were kept within 1LOT for analysis if they switched from sunitinib to pazopanib (or vice versa). Start date, clinical characteristics and risk scores pertaining to the regimen that they were switched from were assigned to the LOT in question, while discontinuation date (if applicable) and outcomes were related to the regimen the patient was subsequently treated with. Discontinuation of the switched-to regimen defined the end of 1LOT. Patients who proceeded to receive other treatment were considered as moving onto a subsequent LOT.

MSKCC scores [11, 12] at 1LOT and 2 + LOT were calculated post-study and a point accrued for matching each of the following criteria: 1) Karnosfky PS < 80; 2) calcium ≥2.5 mmol/L; 3) haemoglobin < 13.5/12.0 g/dL (male/female); 4) (1LOT only) days since RCC diagnosis ≤365; 5) (1LOT only) lactate dehydrogenase (LDH) > 1.5 times the upper limit of normal (i.e. 369 IU/L and 825 IU/L for Cambridge and Manchester, respectively). Risk classifications for 1LOT/2 + LOT were: Favourable: 0/0; Intermediate: 1–2/1; Poor: 3–5/2–3.

### Statistical analysis

Baseline patient demographic and clinical characteristics were analysed descriptively. There was no specific research hypothesis pertaining to OS and therefore no formal sample size calculations were required with respect to statistical power. OS estimates and other time-to-event variables were determined using Cox multivariate proportional hazards models and Kaplan-Meier analysis methods with log-rank and Wilcoxon tests used to compare outcomes, with significance level defined as *p* < 0.05. Confidence interval (CI) was calculated using the Wilson Score method for estimated proportions. All statistical analyses were performed using R (version 3.4.2). Kaplan-Meier curves were generated using R’s survival package.

## Results

### Patient population

Of the 840 patients who met the inclusion criteria, 188 were excluded because they satisfied one or more of the following exclusion criteria: not treated exclusively with NICE/CDF-recommended systemic therapies (*n* = 120, 67% of which received IL-2 or IFN-α therapy); participated in a clinical trial in which systemic therapies were not administered within SoC (*n* = 72); or were LTFU prior to 30 June 2016 (*n* = 29). Data from 652 patients were included in the final analysis, with 424 (65.0%) and 228 (35.0%) patients from Manchester and Cambridge respectively. Baseline characteristics are presented in Table 1, patients were on average 64.8 years old, predominantly male (65.3%) and white (96.5%). 79.5% had clear-cell mRCC subtype, 30.7% of patients were hypertensive and 12.0% had type 2 diabetes mellitus. The percentage of patients with favourable, intermediate and poor MSKCC scores at 1LOT were 11.2, 58.3 and 26.7% respectively.

**Table 1:** Baseline characteristics of patients included in the study

Characteristic	Patients receiving 1LOT	Patients receiving 2LOT	Patients receiving 3LOT
Number of Patients	652	184	18
Centre			
Cambridge	228 (35.0%)	58 (31.5%)	5 (27.8%)
Manchester	424 (65.0%)	126 (68.5%)	13 (72.2%)
Age at index, mean (SD)	64.84 (10.5)	62.97 (10.3)	65.06 (8.9)
< 70 years, *n* (%)	424 (65.0%)	130 (70.7%)	13 (72.2%)
≥ 70 years, *n* (%)	228 (35.0%)	54 (29.4%)	5 (27.8%)
Male, *n* (%)	426 (65.3%)	124 (67.4%)	14 (77.8%)
Weight (kg), mean (SD)	78.12 (17.5)	79.99 (17.8)	77.92 (12.6)
Missing, *n* (%)	243 (37.3%)	57 (31.0%)	6 (33.3%)
Histological subtype, *n* (%)			
Clear cell	518 (79.5%)	141 (76.6%)	13 (72.2%)
Non-clear cell	70 (10.7%)	28 (15.2%)	4 (22.2%)
Other	22 (3.4%)	5 (2.7%)	1 (5.6%)
MSKCC prognostic risk score (at time of LOT initiation), *n*(%)			
Favourable	73 (11.2%)	27 (14.7%)	2 (11.1%)
Intermediate	380 (58.3%)	77 (41.9%)	11 (61.1%)
Poor	174 (26.7%)	59 (32.1%)	2 (11.1%)
Missing	25 (3.8%)	21 (11.4%)	
Ethnicity, *n* (%)			
White	629 (96.5%)	176 (95.7%)	17 (94.4%)
Black	4 (0.6%)	2 (1.1%)	0 (0.0%)
Asian (Chinese/other)	1 (0.2%)	1 (0.5%)	0 (0.0%)
Asian (South-East Asian)	15 (2.3%)	5 (2.7%)	1 (5.6%)
Mixed	0 (0.0%)	0 (0.0%)	0 (0.0%)
Other	3 (0.5%)	0 (0.0%)	0 (0.0%)
Comorbidity (History of), *n* (%)			
Hypertension	200 (30.7%)	55 (29.9%)	8 (44.4%)
T1DM	5 (0.8%)	1 (0.5%)	0 (0.0%)
T2DM	78 (12.0%)	27 (14.7%)	0 (0.0%)
CAD	29 (4.5%)	4 (2.2%)	0 (0.0%)
Congestive HF	4 (0.6%)	0 (0.0%)	0 (0.0%)
Hypothyroidism	21 (3.2%)	6 (3.3%)	1 (5.6%)
VTE	6 (0.9%)	1 (0.5%)	0 (0.0%)
Autoimmune disease	1 (0.2%)	0 (0.0%)	0 (0.0%)
Conditions involving regular corticosteroid therapy	5 (0.78%)	0 (0.0%)	0 (0.0%)
Other	229 (35.1%)	62 (33.7%)	5 (27.8%)

*CAD* Coronary Artery Disease, *ECOG PS* Eastern Cooperative Oncology Group Performance Status, *HF* Heart failure, *MSKCC* Memorial Sloan-Kettering Cancer Center, *SD* Standard deviation, *T1DM* Type 1 diabetes mellitus, *T2DM* Type 2 diabetes mellitus, *VTE* Venus Thromboembolism

### Treatment patterns

All 652 included patients received treatment at 1LOT, with the majority receiving sunitinib (60.7%) or pazopanib (37.7%; Table 2). 8.9% of patients switched regimens (5.7% from pazopanib to sunitinib and 3.2% from sunitinib to pazopanib; Supplementary Fig. 1, Additional File 1). One hundred eighty-four patients received treatment at 2LOT, with the majority receiving axitinib (57.1%) or everolimus (41.9%). Eighteen patients received treatment at 3LOT; 72.2% received everolimus and 22.2% received axitinib. No patients received treatment at 4LOT. Mean (95% CI) follow up was 23.8 (22.2–25.4) months. 21.5, 26.1 and 33.3% of patients remained on 1LOT, 2LOT and 3LOT treatments at follow-up respectively. Time to events by LOT and adverse events by LOT are summarised in Supplementary Table 1 and 2 (Additional File 1) respectively.

**Table 2:** Treatments received by LOT

Treatment, *n* (%)	1LOT	2LOT	3LOT
Received treatment	652	184	18
Sunitinib	396 (60.7%)	1 (0.5%)	–
Originally prescribed	375 (57.5%)	1 (0.5%)	–
Pazopanib intolerant	21 (3.2%)	–	–
Pazopanib	246 (37.7%)	–	–
Originally prescribed	209 (32.1%)	–	–
Sunitinib intolerant	37 (5.7%)	–	–
Axitinib	–	105 (57.1%)	4 (22.2%)
Everolimus	4 (0.6%)	77 (41.9%)	13 (72.2%)
Other	6 (0.9%)	1 (0.5%)	1 (5.6%)
On treatment at FU	140 (21.5%)	48 (26.1%)	6 (33.3%)

*LOT* Line of therapy, *FU* Follow-Up

### Overall survival

**Fig. 1:**
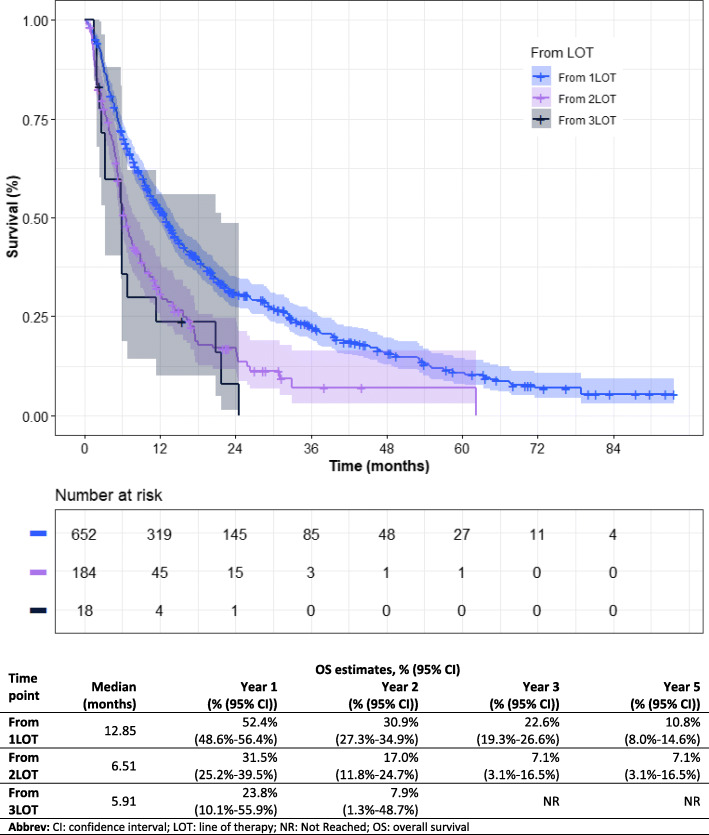
Overall survival by LOT. Shaded region denotes 95% confidence interval

Median OS for all patients who received a 1LOT was 12.9 months from initiation, with a one-year survival estimate of 52.4% (95% CI: 48.6–56.4%; Fig. 1). Median OS from 1LOT initiation increased to 20.8 months and 36.7 months for those who received 2 + LOTs and 3LOTs respectively. For those patients who received a 2LOT, median OS from 2LOT initiation was 6.5 months with a one-year survival estimate of 31.5% (95% CI: 25.2–39.5%)). Median OS from 3LOT initiation was 5.9 months with a one-year survival estimate of 23.8% (95% CI: 10.1–55.9%). Median OS from initiation of 1LOT of the 89 excluded patients who received either IL-2 or IFN-α at any point during the study was 47.5 months (Supplemental Fig. S3).

**Fig. 2:**
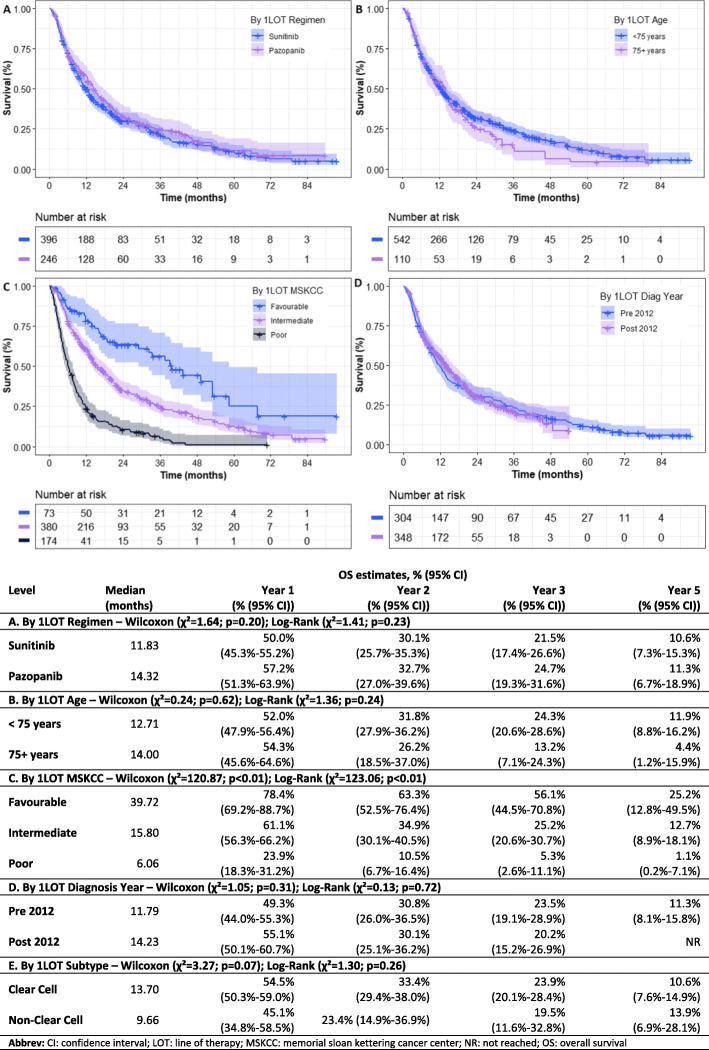
Overall survival from 1LOT stratified by key baseline characteristics. **a**: By regimen; **b**: By Age; **c**: By MSKCC score; **d**: By Diagnosis Year; **e**: By Histological Subtype (not shown)

The effects of treatment regimen, age, MSKCC classification (at treatment initiation), diagnosis date (pre 2012 (01 January 2008 to 31 December 2011)/post 2012 (01 January 2012 to 31 December 2015)), and histological subtype on OS for 1LOT and 2LOT are reported in Fig. 2 and Supplementary Fig. 2 (Additional File 1) respectively. In both 1LOT and 2LOT, significant differences were observed between OS and MSKCC classification (*p* < 0.001). At both LOTs, favourable-risk patients achieved the best survival outcomes (median OS; 1LOT – 39.7 months; 2LOT – 14.3 months), compared with intermediate-risk (median OS; 1LOT – 15.8 months; 2LOT – 8.9 months) and poor-risk patients (median OS; 1LOT – 6.1 months; 2LOT – 3.3 months). Furthermore, OS was higher in patients treated between 2012 and 15 (14.2 months) comparted with those treated between 2008 and 11 (11.8 months). To validate inferences made, a Cox multivariate proportional hazards model was implemented at both 1LOT and 2LOT. Inferences were consistent across the two approaches.

## Discussion

This retrospective, observational chart review study aimed to determine the real-world effectiveness of common targeted systemic therapies for patients with mRCC within routine UK clinical practice. Baseline characteristics of the study population were comparable to the demographics of the UK renal cancer population in terms of sex and the proportion of patients with clear cell mRCC [1], however our cohort were notably younger than the average UK kidney cancer population. In this study, only 35% of patients were aged 70 and older, whereas UK statistics indicate that approximately half of newly diagnosed patients are ≥70 years old [1]. This may be because the focus of this study was on those newly diagnosed with mRCC (as opposed to non-metastatic RCC) and was limited to patients treated with targeted systemic therapies.

During the study period (2008–2015), NICE technical advisory guidance outlined recommendations for sunitinib (TA169) and pazopanib (TA215) at first line from 2009 and 2011 respectively, and for everolimus (TA219) and axitinib (TA333) at second line from 2011 and 2015 respectively [14]. Treatment pathways agreed by NICE indicate that approved 2LOT therapies are also used for 3LOT [15]. Data were analysed in this study to align with the NICE pathways for advanced and/or metastatic RCC, therefore the received treatments were consistent with NICE-recommended systemic therapies. The majority of patients in this study received sunitinib or pazopanib at 1LOT (98.5%) and everolimus and axitinib at 2LOT (99.0%) and 3LOT (94.4%).

Only 28% of 1LOT patients received a 2LOT, and only 3% of 1LOT patients received a 3LOT. Figures published more recently (2012–2016) by one of the sites involved in this study are 48 and 16% respectively [16], which likely reflect the lower numbers of favourable prognosis patients included in this study and an increase in available treatments over time. Indeed, since the beginning of the study period, several newer therapies for mRCC patients have been approved (i.e. tivozanib (TA512), cabozantinib (TA542) and nivolumab with ipilimumab (TA581) at 1LOT; lenvatinib with everolimus (TA498), cabozantinib (TA463), and nivolumab (TA417) at 2 + LOT) [14]. Furthermore, no patients received a 4LOT treatment, which may reflect the fact that no fourth-line treatments have been approved by NICE, therefore limiting the therapeutic options for mRCC patients post-3LOT.

Both 1-year and 5-year survival within the study period (2008–2015) were higher than the available estimates for England (from 2013 to 2015; 1 year: 52.4% vs 37.5% [17] and 5-year: 10.8% vs 5.2–6.6% [18]), and comparable to a Swedish real-world study over a similar period [19]. However, the OS observed in this study was lower than that reported in an earlier retrospective multi-centre UK database study (Renal Cell Carcinoma Outcomes Research Dataset, RECCORD) [20], and in real-world studies from other countries over a similar timeframe [21–24]. The RECCORD study included only patients with clear cell renal cancer (80% of our cohort), and included patients on clinical trials and a small number of patients receiving IL-2 or IFN-α. Median age was also younger (61 years). Subgroup analyses (Supplementary Fig. 3, Additional File 1) of the 89 patients excluded from the main analysis because they received IL-2 or IFN-α at any point during the study revealed a median OS of almost four-times longer (47.5 vs. 12.9 months at 1LOT) than patients treated exclusively with NICE/CDF-recommended systemic therapies and reflects the fact that the Manchester Centre is a national treatment centre for high-dose IL-2 which, in carefully selected patients, can have an excellent outcome [25]. In addition, a further 72 patients were excluded because they participated in clinical trials where systemic therapies were not administered within standard of care. These patients were excluded as they would have biased OS in favour of better outcomes and may partly explain the shorter OS observed in this analysis compared with similar studies.

Survival prognoses within the study were strongly linked with MSKCC risk score. This method is a frequently utilised [26] and validated [27, 28] scoring system for prediction of patient survival and was reliable in this cohort of mRCC patients. Favourable-risk patients achieved a 6.6-fold increase in OS (compared with poor-risk patients) at 1LOT and a 4.3-fold increase at 2LOT. Although this association remained in 1LOT patients, the improvement was somewhat diminished from 18 months onwards in the 2LOT cohort, probably reflecting the lack of suitable subsequent approved therapies within this disease area. It is however important to note that all analyses are descriptive in nature and confounding variables not controlled for. In this study, the proportion of patients in the favourable risk category was lower (11% vs 25%) and poor risk patients slightly higher (27% vs 22%) compared with a reference mRCC population [11]. This finding is likely due to variations in the characteristics of the study populations and our exclusion of patients receiving cytokine therapies from the main analysis, who had significantly longer OS and more favourable distribution of MSKCC scores.

The nature of real-world research means there is heterogeneity across treatments used at each LOT and across patient characteristics involved in each study, and therefore comparison between study outcomes should be done with caution. Our study focused on patients for whom a full medical history was available and had one or more treatments, therefore becoming susceptible to immortal time bias. Furthermore, participation was limited to two specialist RCC centres and inclusion was limited to patients who received NICE-recommended targeted systemic therapies alone (not in conjunction with cytokine therapy or within a clinical trial), therefore it is unclear how generalisable the study findings are to alternative patient populations or settings.

Despite the limitations associated with this study, including the large numbers of excluded patients, the study remains the largest real-world study on the treatment patterns and outcomes of mRCC patients in the UK. The study was limited to an assessment of the treatment patterns and outcomes of patients receiving targeted systemic therapies; however, with increasing use of immunotherapy in recent years, the impact of this group of therapies in the treatment of mRCC warrants investigation in future studies.

## Conclusions

In this real-world study, treatment was consistent with current NICE guidelines for mRCC patients. Although the study population was selected in favour of poorer prognosis patients, and median OS was lower than that that reported by a UK database study over a similar period, both 1-year and 5-year survival outcomes in this study were more favourable than those reported by other studies in England at the same time. Overall, survival outcomes in this ‘real world’ population remain poor and indicates significant unmet need for effective and safe treatment options which could improve survival among mRCC patients.

## Supplementary information

**Additional file 1: Supplementary Table 1.** Time-to-events by LOT**. Supplementary Table 2.** Adverse events by LOT. **Supplementary Figure 1.** Treatment pathways for patients receiving systemic therapies. **Supplementary Figure 2.** Overall survival from 2LOT stratified by key baseline characteristics. **Supplementary Figure 3.** Overall survival by LOT in patients receiving interleukin-2/interferon-alpha at any LOT

## Data Availability

The data that support the findings of this study are available from Bristol Myers Squibb Pharmaceuticals Ltd., but restrictions apply to the availability of these data, which were used under license for the current study, and so are not publicly available. Data are however available from the authors upon reasonable request and with permission of Bristol Myers Squibb Pharmaceuticals Ltd.

## Abbreviations

AEs: Adverse events
CDF: Cancer Drug Fund
CI: Confidence interval
ECOG: Eastern Cooperative Oncology Group
eCRF: Electronic case report form
GPP: Good pharmacology practice
IFN-α: Interferon-alpha
IL-2: Interleukin-2
ISPE: International Society for Pharmacoepidemiology
LDH: Lactate dehydrogenase
LOT: Line of therapy
LTFU: Lost to follow-up
mRCC: Metastatic renal cell carcinoma
MSKCC: Memorial Sloan Kettering Cancer Centre
NICE: National Institute for Health and Care Excellence
OS: Overall survival
PFS: Progression free survival
PS: Performance score
RCC: Renal cell carcinoma
SoC: Standard of care
UK: United Kingdom
