# Supplementary material for: Treatment patterns and health outcomes in metastatic renal cell carcinoma patients treated with targeted systemic therapies in the UK
Source: BMC Cancer. 2020 Jul 17;20:670. doi: 10.1186/s12885-020-07154-z (PMC7368711; doi:10.1186/s12885-020-07154-z)
Supplement: Supplementary file 1 — Additional file 1: Supplementary Table 1. Time-to-events by LOT. Supplementary Table 2. Adverse events by LOT. Supplementary Figure 1. Treatment pathways for patients receiving systemic therapies. Supplementary Figure 2. Overall survival from 2LOT stratified by key baseline characteristics. Supplementary Figure 3. Overall survival by LOT in patients receiving interleukin-2/interferon-alpha at any LOT [file 12885_2020_7154_MOESM1_ESM.docx]

Supplementary Table 1. Time-to-events by LOT

|  | **1LOT**  **(n = 652)** | **2LOT**  **(n = 184)** | **3LOT**  **(n = 18)** |
| --- | --- | --- | --- |
| **Treatment initiation**† |  |  |  |
| Mean time (months) to treatment initiation (95% CI) | 6.5 (5.6-7.3) | 1.2 (0.8-1.6) | 1.0 (0.5-1.5) |
| **Treatment holiday** |  |  |  |
| n patients (%) | 339 (52.0%) | 80 (43.5%) | 6 (33.3%) |
| Mean duration (days) of treatment holiday* (95% CI) | 54.4 (43.5-65.4) | 35.2 (20.5-49.8) | 20.3 (1.3-39.3) |
| **Dose increase or reduction** |  |  |  |
| n patients (%) | 298 (45.7%) | 82 (44.6%) | 4 (22.2%) |
| Mean time (months) to dose increase or reduction* (95% CI) | 4.8 (3.8-5.8) | 2.1 (1.4-2.9) | 2.1 (1.3-3.0) |
| **Discontinuation** |  |  |  |
| n patients (%) | 574 (88.0%) | 159 (86.4%) | 16 (88.9%) |
| Mean time (months) to discontinuation* (95% CI) | 10.5 (9.5-11.6) | 5.2 (4.2-6.3) | 5.6 (1.7-9.5) |
| **Reason for discontinuation, n (%)** |  |  |  |
| Disease progression | 411 (71.6%) | 115 (72.3%) | 11 (68.8%) |
| Treatment toxicity/adverse event | 108 (18.8%) | 31 (19.5%) | 5 (31.3%) |
| Other | 106 (18.5%) | 33 (20.8%) | 2 (12.5%) |
| **Abbrev:** LOT: line of therapy; CI: confidence interval  *Mean time to/of event amongst patients that experienced event  †Calculated as the time from index date or end date or previous LOT to start date of subsequent LOT  NB: more than one reason for discontinuation is possible hence the number of patients may exceed total that experience discontinuation | | | |

**Supplementary Table 2.** Adverse events by LOT

| **Adverse Event, n (%)** | **1LOT** | **2LOT** | **3LOT** |
| --- | --- | --- | --- |
| **Number of Patients** | 652 | 184 | 18 |
| Fatigue | 411 (63.0%) | 103 (56.0%) | 8 (44.4%) |
| Sore Mouth | 309 (47.4%) | 73 (39.7%) | 8 (44.4%) |
| Diarrhoea | 284 (43.6%) | 66 (35.9%) | 5 (27.8%) |
| Nausea | 251 (38.5%) | 40 (21.7%) | 4 (22.2%) |
| Hypertension | 202 (31.0%) | 25 (13.6%) | 2 (11.1%) |
| Hand Foot Syndrome | 148 (22.7%) | 31 (16.8%) | 1 (5.6%) |
| Dyspnoea | 134 (20.6%) | 51 (27.7%) | 5 (27.8%) |
| Vomiting | 121 (18.6%) | 20 (10.9%) | 2 (11.1%) |
| Weight Loss | 117 (17.9%) | 23 (12.5%) | 2 (11.1%) |
| Skin Rash | 94 (14.4%) | 28 (15.2%) | 0 (0.0%) |
| Anaemia | 85 (13.0%) | 25 (13.6%) | 4 (22.2%) |
| Hypothyroidism | 70 (10.7%) | 14 (7.6%) | 3 (16.7%) |
| Fever | 35 (5.4%) | 4 (2.2%) | 1 (5.6%) |
| Psychological | 34 (5.2%) | 5 (2.7%) | 2 (11.1%) |
| Fluid Retention | 34 (5.2%) | 11 (6.0%) | 1 (5.6%) |
| Reduced Renal Function | 33 (5.1%) | 11 (6.0%) | 0 (0.0%) |
| Thrombocytopenia | 33 (5.1%) | 1 (0.5%) | 1 (5.6%) |
| Joint Muscle Problems | 31 (4.8%) | 6 (3.3%) | 0 (0.0%) |
| Impaired Wound Healing | 29 (4.4%) | 6 (3.3%) | 0 (0.0%) |
| Irregular Heart Beat | 17 (2.6%) | 4 (2.2%) | 0 (0.0%) |
| Hyperthyroidism | 4 (0.6%) | 1 (0.5%) | 0 (0.0%) |
| Hypotension | 3 (0.5%) | 1 (0.5%) | 0 (0.0%) |
| Pneumonitis | 1 (0.2%) | 6 (3.3%) | 0 (0.0%) |
| Myocarditis | 0 (0.0%) | 0 (0.0%) | 0 (0.0%) |
| **Abbrev:** LOT: Line of therapy; FU: Follow-Up; | | | |


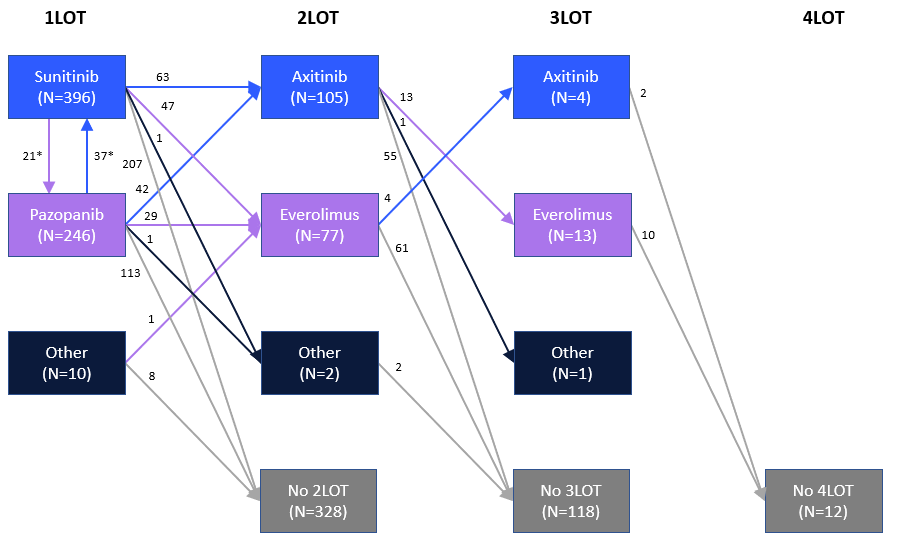


**Supplementary** **Figure 1.** Treatment pathways for patients receiving systemic therapies
** Denotes patients who were transferred to a supplementary 1LOT regimen due to intolerance of the exiting regimen.*

**
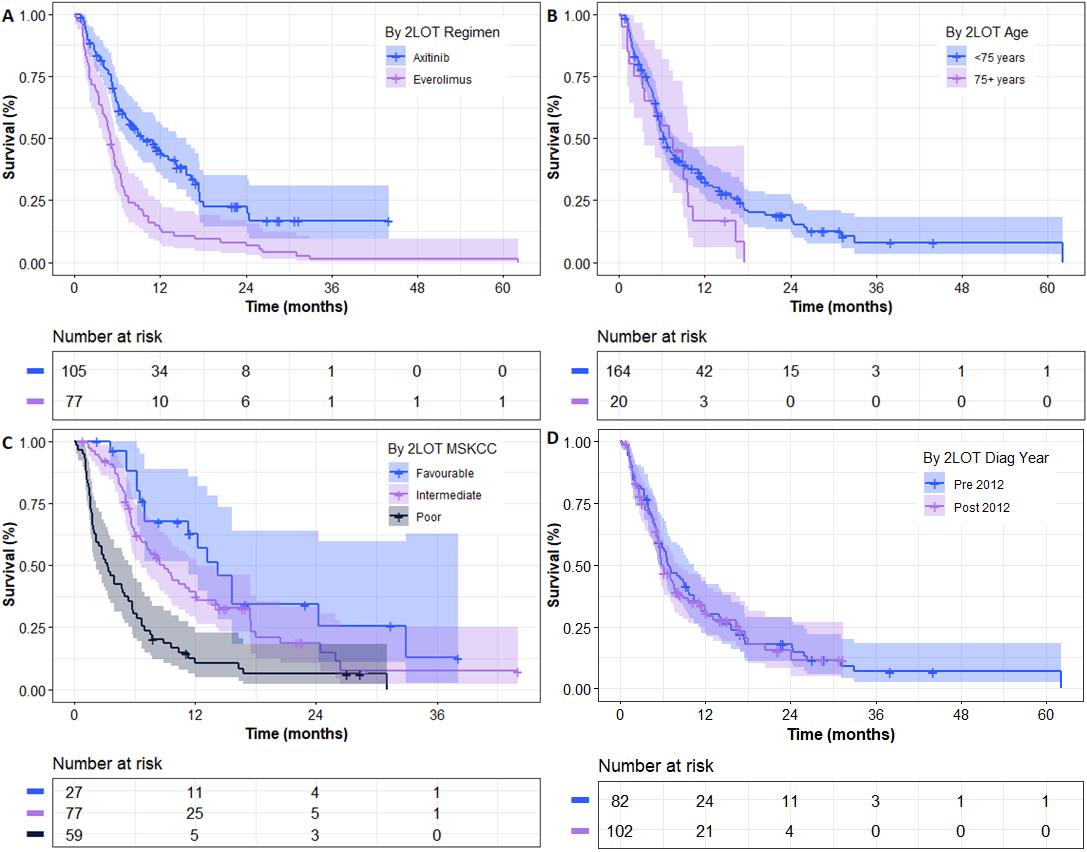
**

| **Level** | **OS estimates, % (95% CI)** | | | | |
| --- | --- | --- | --- | --- | --- |
|  | **Median (months)** | **Year 1 (% (95% CI))** | **Year 2 (% (95% CI))** | **Year 3 (% (95% CI))** | **Year 5 (% (95% CI))** |
| **A. By 2LOT Regimen – Wilcoxon (χ²=20.77; p<0.01); Log-Rank (χ²=21.56; p<0.01);** | | | | | |
| **Axitinib** | 9.59 | 45.4%  (36.3%-56.8%) | 22.6%  (14.5%-35.2%) | 16.9%  (9.3%-30.8%) | NR |
| **Everolimus** | 5.03 | 13.4%  (7.5%-23.7%) | 8.0%  (3.7%-17.2%) | 1.3%  (0.2%-9.4%) | 1.3%  (0.2%-9.4%) |
| **B. By 2LOT Age – Wilcoxon (χ²=0.72; p=0.40); Log-Rank (χ²=1.57; p=0.21);** | | | | | |
| **< 75 years** | 6.44 | 33.4%  (26.6%-42.0%) | 19.0%  (13.2%-27.4%) | 7.9%  (3.4%-18.4%) | 7.9%  (3.4%-18.4%) |
| **75+ years** | 7.28 | 16.9%  (6.1%-46.7%) | NR | NR | NR |
| **C. By 2LOT MSKCC – Wilcoxon (χ²=46.11; p<0.01); Log-Rank (χ²=33.48; p<0.01);** | | | | | |
| **Favourable** | 14.26 | 62.8%  (46.1%-85.6%) | 34.3%  (18.4%-63.8%) | 12.9%  (2.5%-65.0%) | NR |
| **Intermediate** | 8.90 | 39.3%  (29.2%-52.7%) | 18.7%  (10.6%-33.0%) | 7.5%  (2.2%-25.2%) | NR |
| **Poor** | 3.32 | 10.6%  (4.9%-23.0%) | 6.3%  (2.2%-18.2%) | NR | NR |
| **D. By 2LOT Diagnosis Year – Wilcoxon (χ²=0.13; p=0.72); Log-Rank (χ²=0.07; p=0.79)** | | | | | |
| **Pre 2012** | 6.70 | 31.3%  (22.6%-43.4%) | 18.0%  (11.2%-29.1%) | 6.9% (2.6%-18.1%) | 6.9% (2.6%-18.1%) |
| **Post 2012** | 6.05 | 32.2%  (23.8%-43.7%) | 15.4%  (8.2%-28.9%) | NR | NR |
| **E. By 2LOT Subtype – Wilcoxon (χ²=0.00; p=0.98); Log-Rank (χ²=0.02; p=0.90);** | | | | | |
| **Clear Cell** | 6.93 | 33.8%  (26.5%-43.0%) | 17.2%  (11.3%-26.2%) | 11.5%  (6.4%-20.5%) | 11.5%  (6.4%-20.5%) |
| **Non-Clear Cell** | 6.51 | 29.3%  (15.7%-54.8%) | 23.4%  (10.9%-50.4%) | NR | NR |
| **Abbrev:** CI: confidence interval; LOT: line of therapy; MSKCC: memorial sloan kettering cancer center; NR: not reached; OS: overall survival | | | | | |

**Supplementary** **Figure 2.** Overall survival from 2LOT stratified by key baseline characteristics
A: By regimen; B: By Age; C: By MSKCC score; D: By Diagnosis Year; E: By Histological Subtype (not shown)


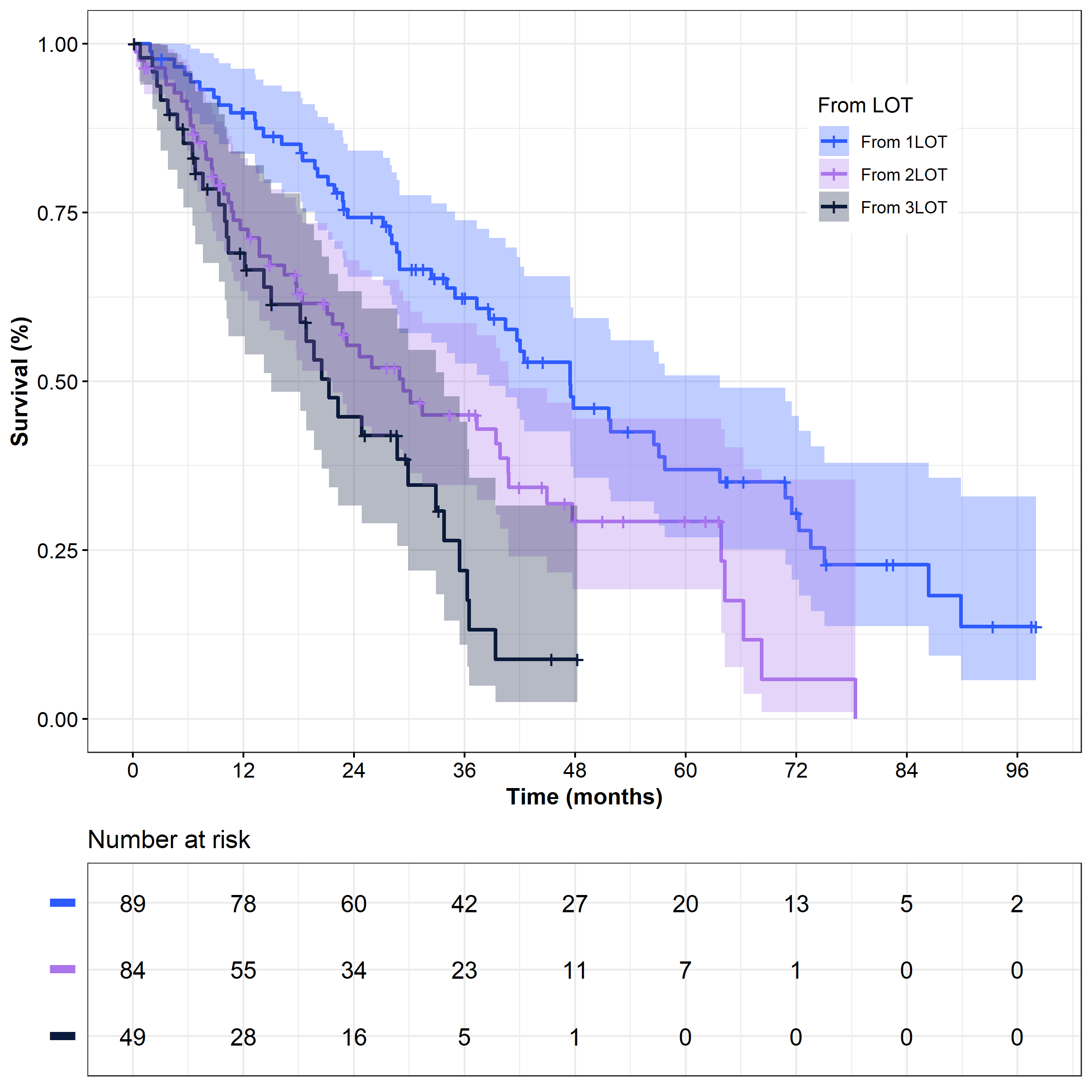


| **Time point** | **OS estimates, % (95% CI)** | | | | |
| --- | --- | --- | --- | --- | --- |
|  | **Median (months)** | **Year 1 (% (95% CI))** | **Year 2 (% (95% CI))** | **Year 3 (% (95% CI))** | **Year 5 (% (95% CI))** |
| **From**  **1LOT** | 47.47 | 89.8%  (83.7%-96.3%) | 74.3%  (65.5%-84.2%) | 62.3%  (52.6%-73.9%) | 37.0%  (26.8%-50.9%) |
| **From**  **2LOT** | 29.34 | 72.5%  (63.4%-83.0%) | 55.3%  (45.0%-67.9%) | 45.0%  (34.6%-58.6%) | 29.2%  (19.2%-44.5%) |
| **From 3LOT** | 21.32 | 69.0%  (56.7%-84.0%) | 44.8%  (31.6%-63.4%) | 22.0%  (11.0%-44.0%) | NR |
| **Abbrev:** CI: confidence interval; LOT: line of therapy; NR: Not Reached; OS: overall survival | | | | | |

**Supplementary Figure 3.** Overall survival by LOT in patients receiving interleukin-2/interferon-alpha at any LOT

Shaded region denotes 95% confidence interval
